# Supplementary material for: A new species of Psychrophrynella (Amphibia, Anura, Craugastoridae) from the humid montane forests of Cusco, eastern slopes of the Peruvian Andes
Source: PeerJ. 2016 Mar 14;4:e1807. doi: 10.7717/peerj.1807 (PMC4793343; doi:10.7717/peerj.1807)
Supplement: Appendix S1 [file peerj-04-1807-s001.docx]

**Appendix 3.** Variation in coloration among collected (holotype and paratypes) and uncollected specimens of *Psychrophrynella chiriampatu* sp. n. Photographs by A. Catenazzi.

| HOLOTYPE (male) | CORBIDI 16495 (AC127.15) |
| --- | --- |
| 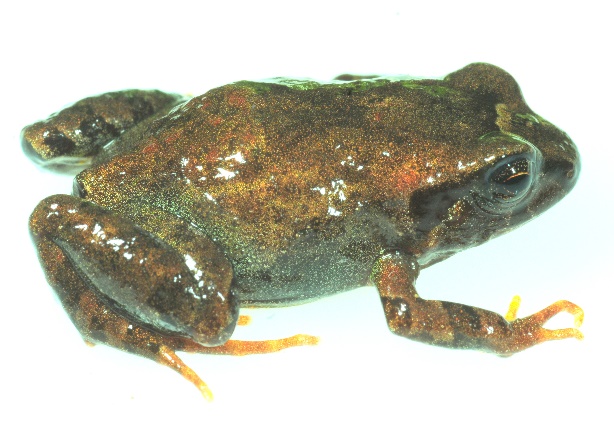 | 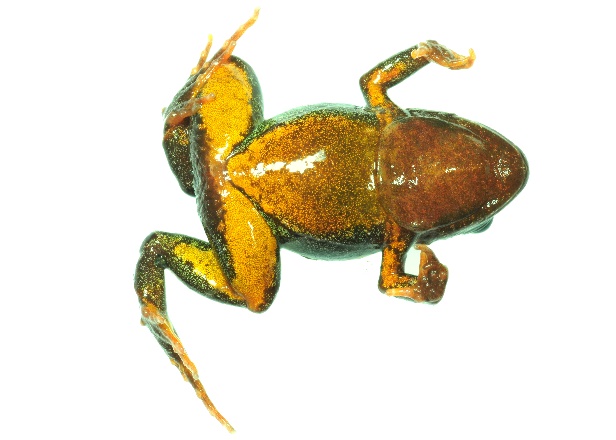 |
| PARATYPE (male) | MHNC 14656 (AC99.15) |
| 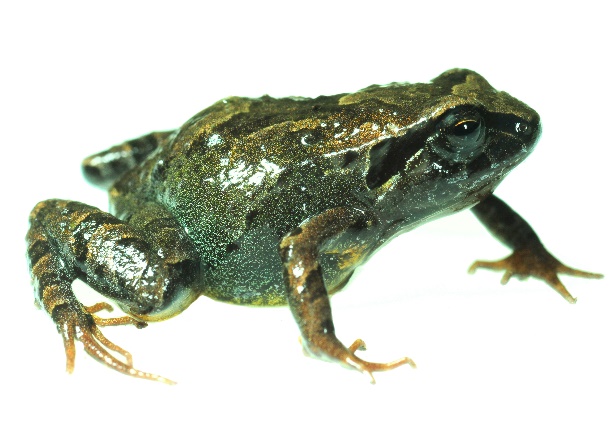 | 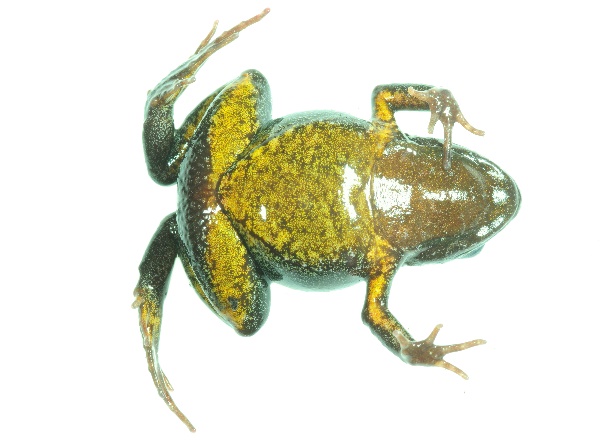 |
| PARATYPE (male) | MHNC 14667 (AC104.15) |
| 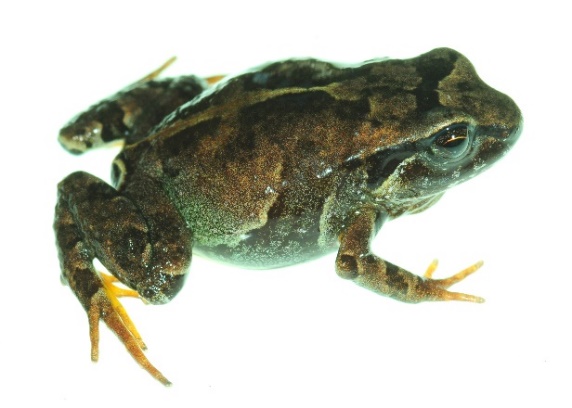 | 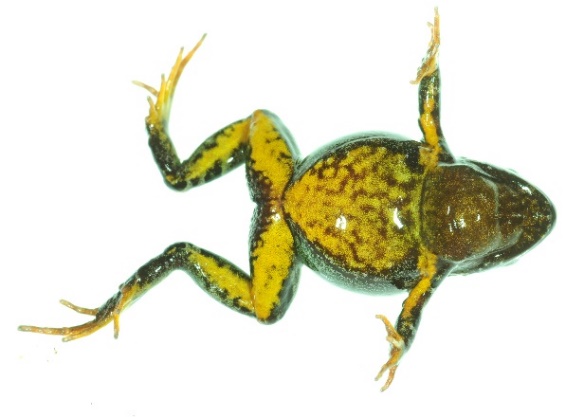 |

| PARATYPE (female) | CORBIDI 16502 (AC 105.15) |
| --- | --- |
| 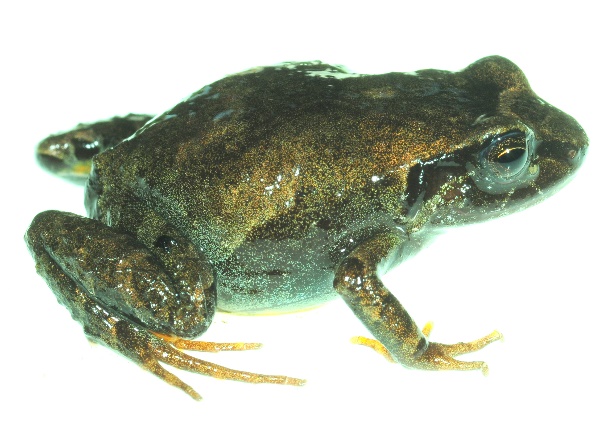 | 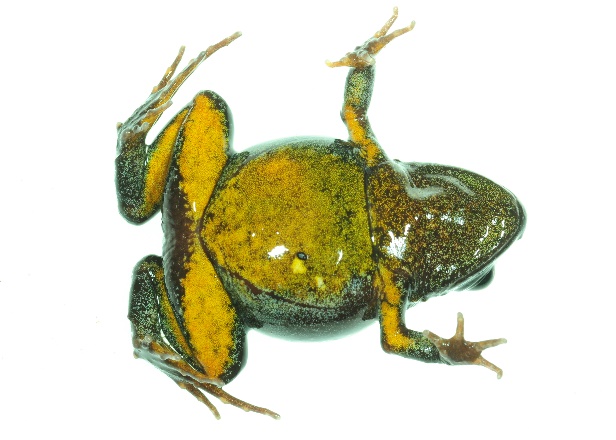 |
| PARATYPE (female) | CORBIDI 16499 (AC 117.15) |
| 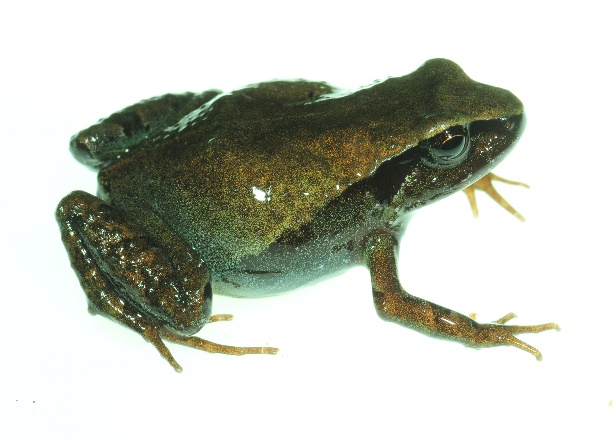 | 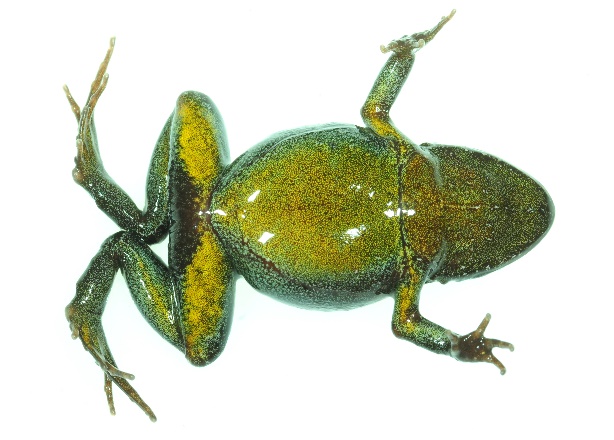 |
| PARATYPE (female) | CORBIDI 16504 (AC86.15) |
| 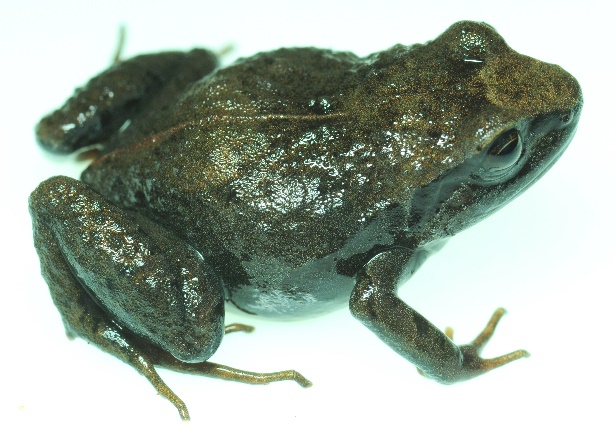 | 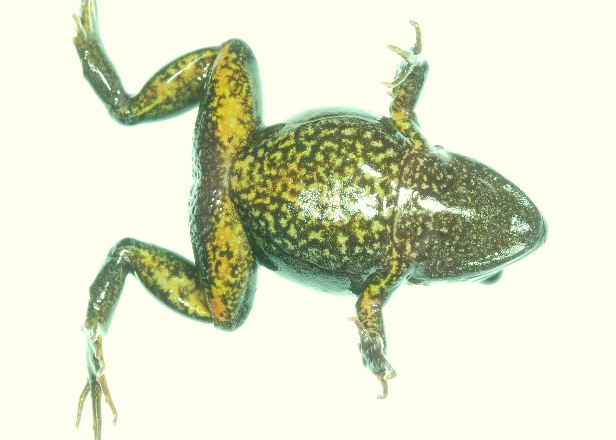 |
| PARATYPE (male) | CORBIDI 16506 (AC87.15) |
| 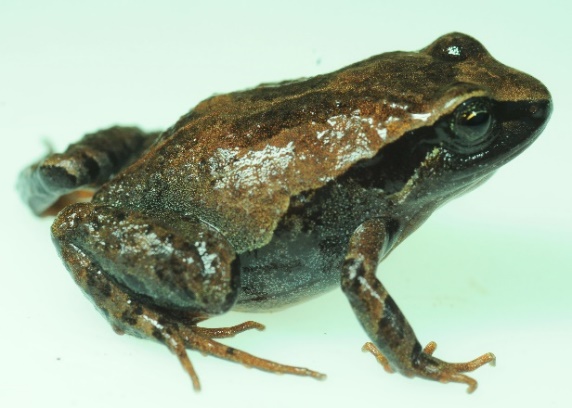 | 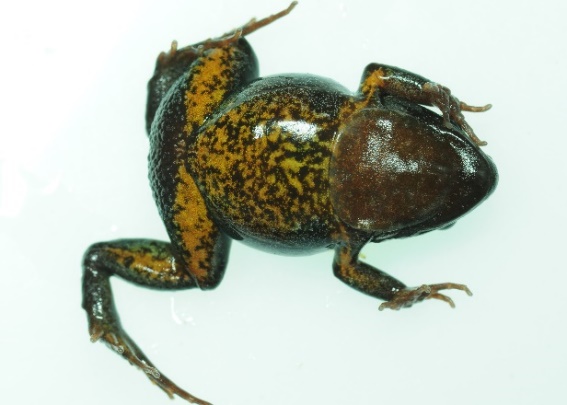 |

.

| PARATYPE (male) | MHNC 14670 (AC88.15) |
| --- | --- |
| 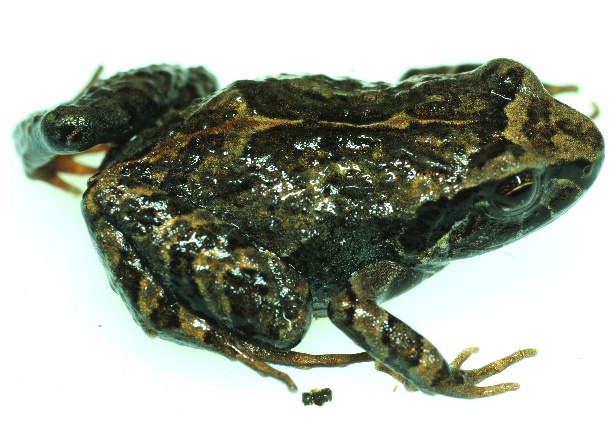 | 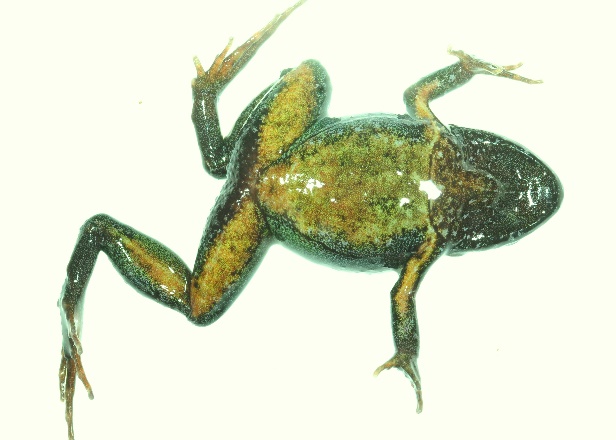 |
| PARATYPE (male) | CORBIDI 16992 (AC89.15) |
| 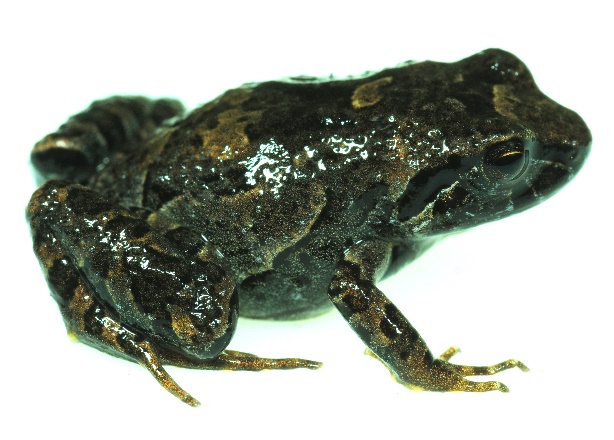 | 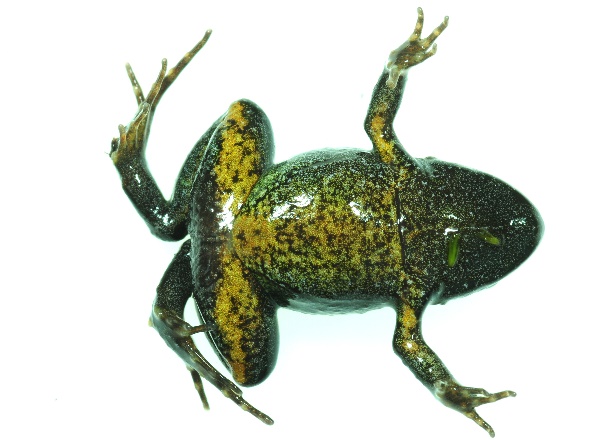 |
| PARATYPE (male) | MHNC 14671 (AC90.15) |
| 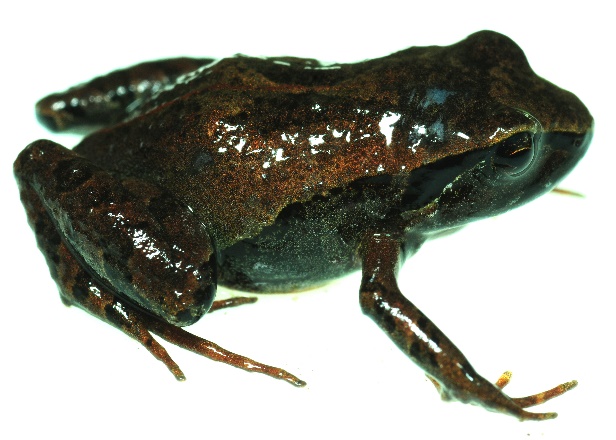 | 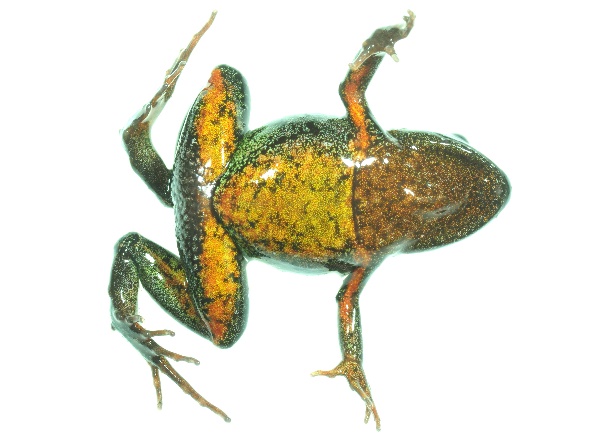 |
| PARATYPE (male) | MHNC 14672 (AC91.15) |
| 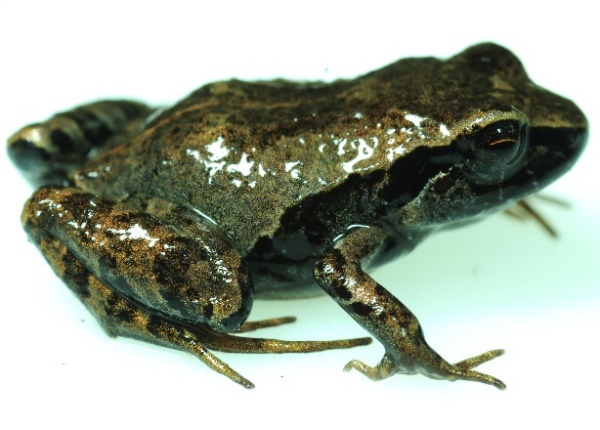 | 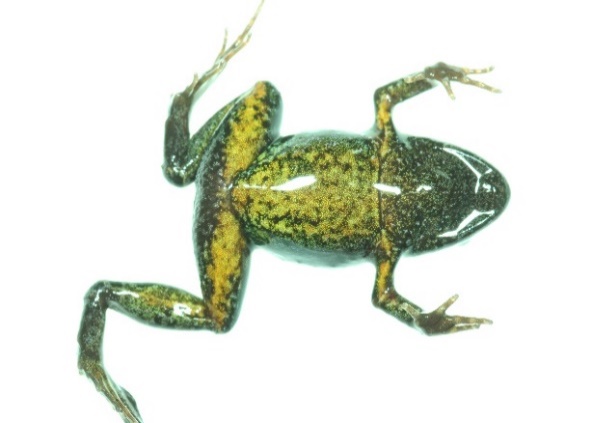 |

.

| PARATYPE (male) | CORBIDI 16993 (AC92.15) |
| --- | --- |
| 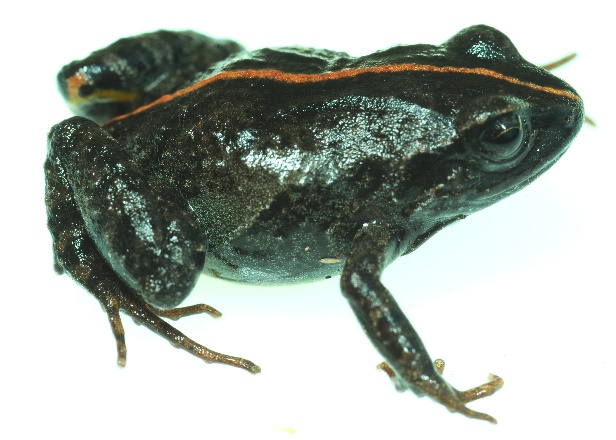 | 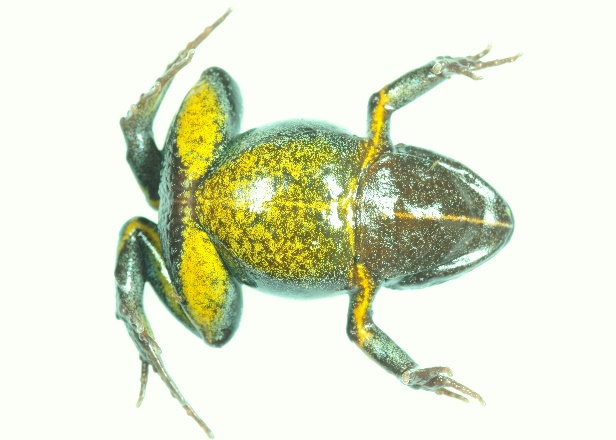 |
| PARATYPE (male) | CORBIDI 16994 (AC93.15) |
| 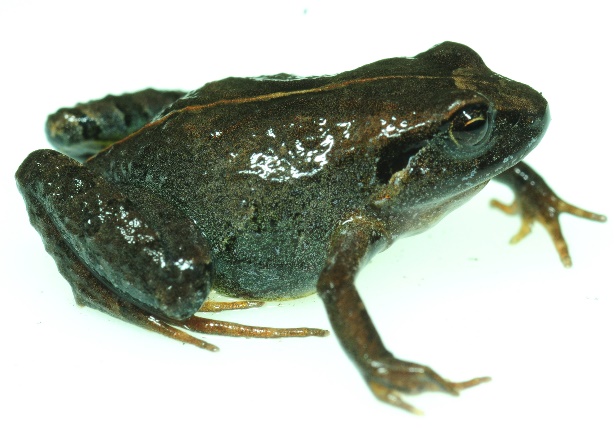 | 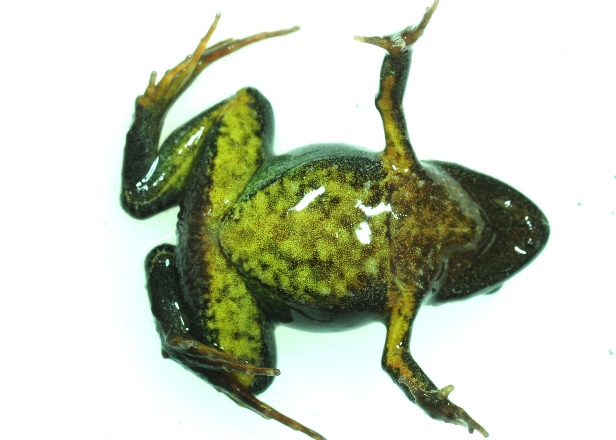 |
| PARATYPE (male) | CORBIDI 16505 (AC100.15) |
| 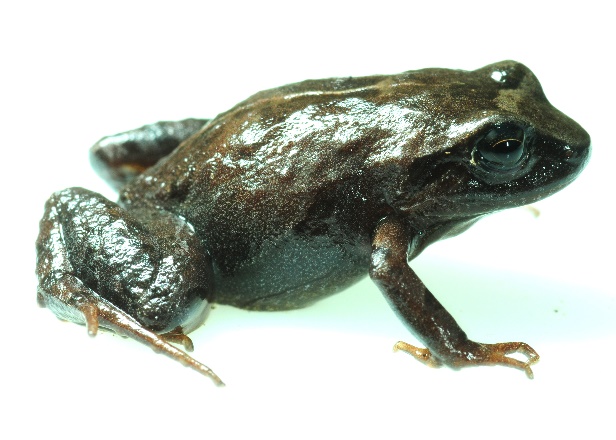 | 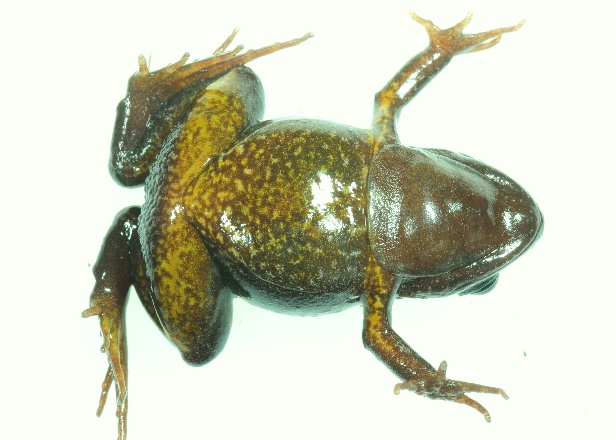 |
| PARATYPE (male) | CORBIDI 16501 (AC102.15) |
| 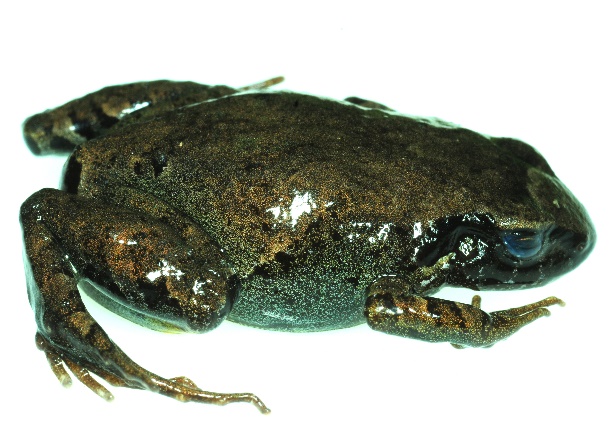 | 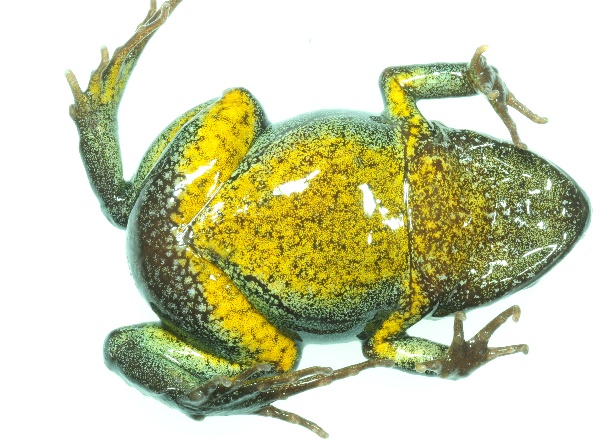 |

.

| PARATYPE (male) | MHNC 14664 (AC103.15) |
| --- | --- |
| 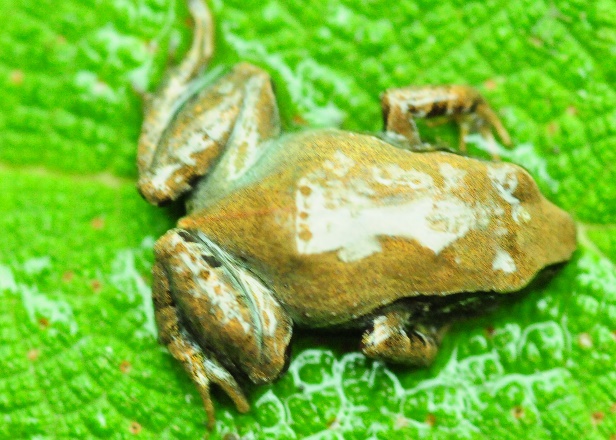 | 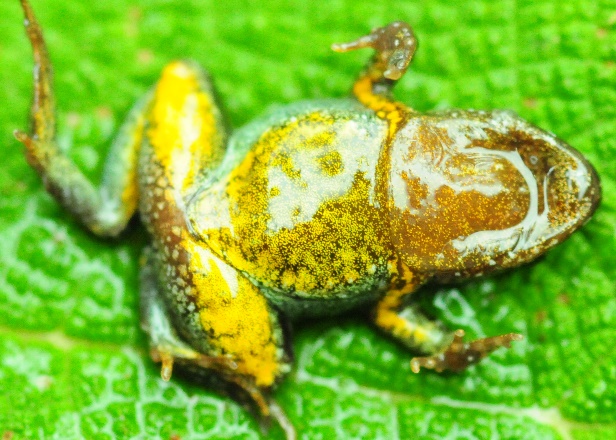 |
| PARATYPE (female) | MHNC 14662 (AC106.15) |
| 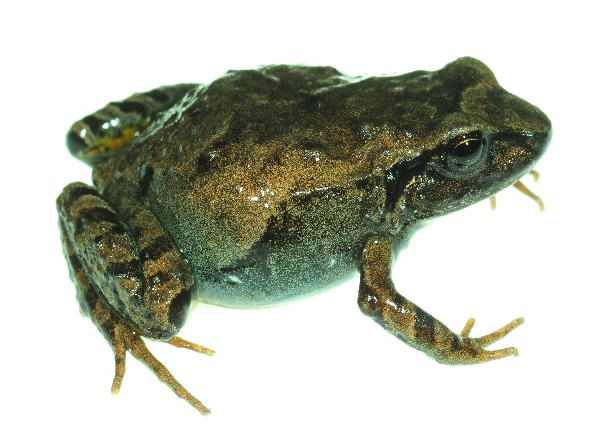 | 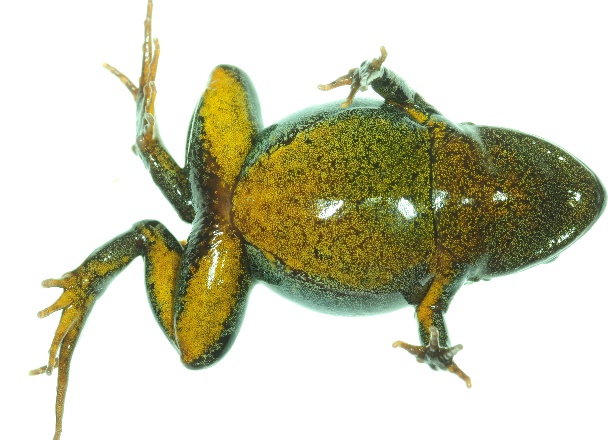 |
| PARATYPE (male) | MHNC 14658 (AC107.15) |
| 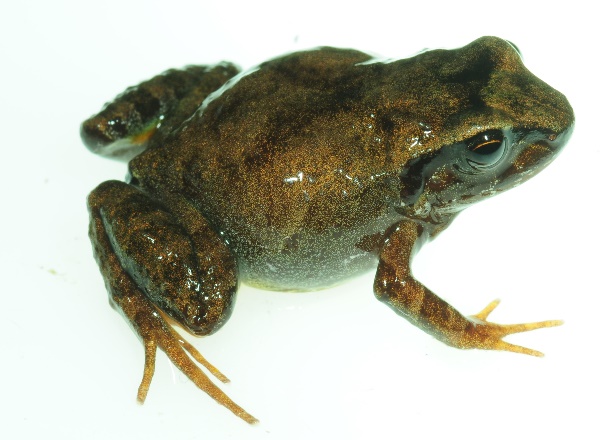 | 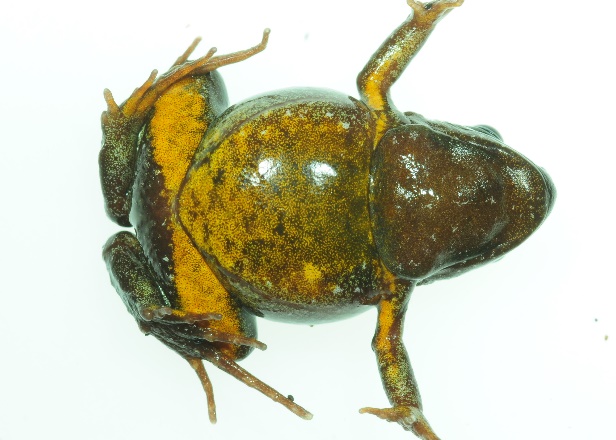 |
| PARATYPE (male) | MHNC 14666 (AC108.15) |
| 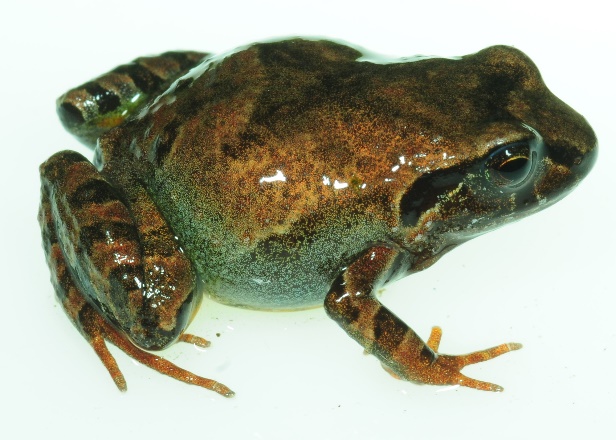 | 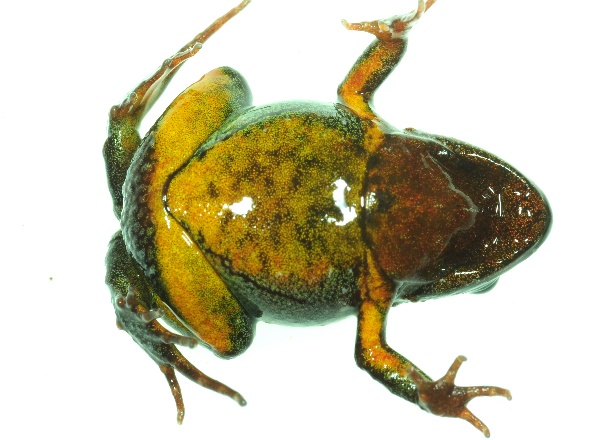 |

.

| PARATYPE (female) | MHNC 14668 (AC110.15) |
| --- | --- |
| 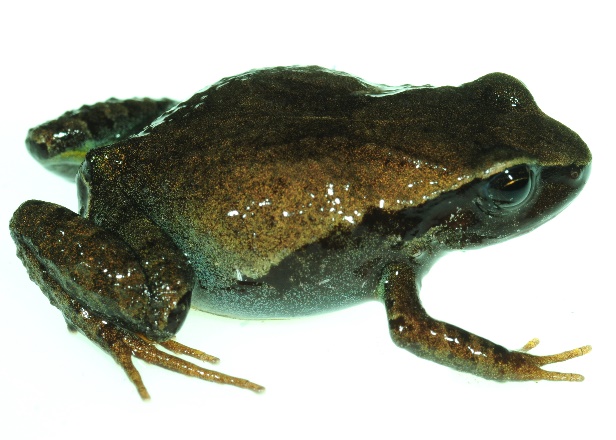 | 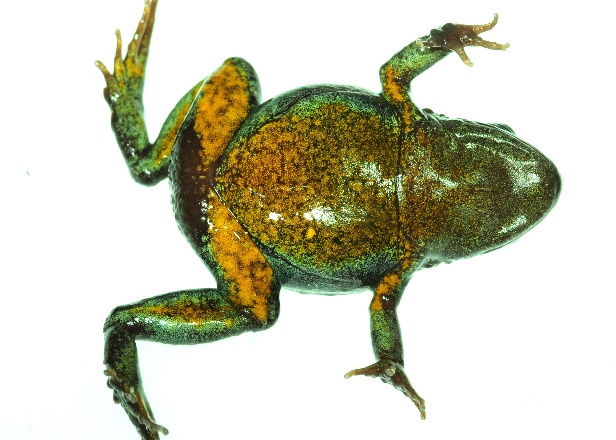 |
| PARATYPE (male) | CORBIDI 16503 (AC111.15) |
| 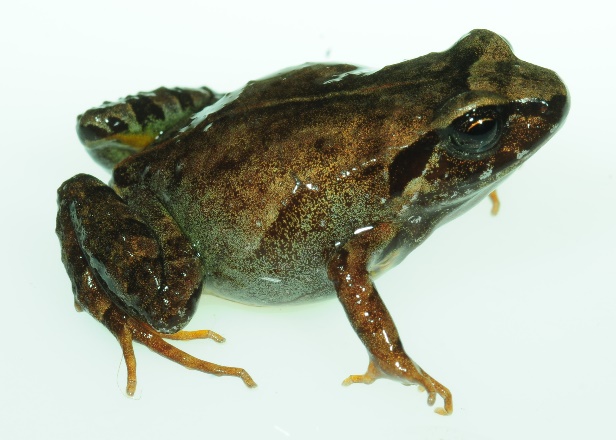 | 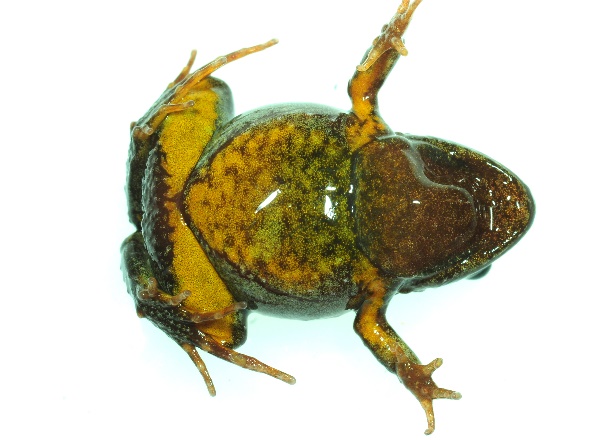 |
| PARATYPE (female) | MHNC 14669 (AC118.15) |
| 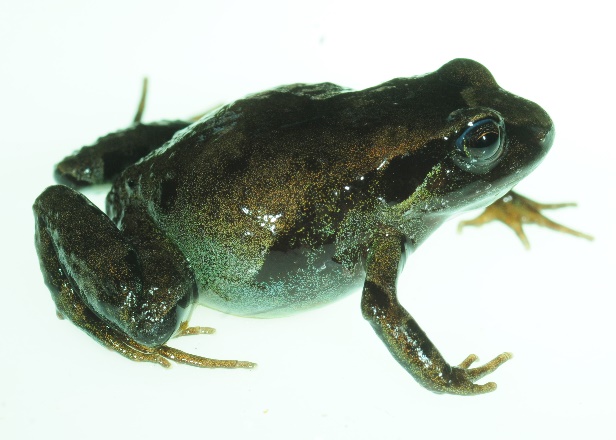 | 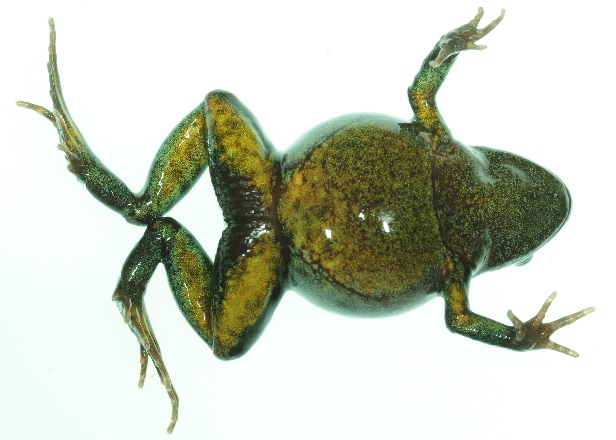 |
| PARATYPE (female) | CORBIDI 16498 (AC119.15) |
| 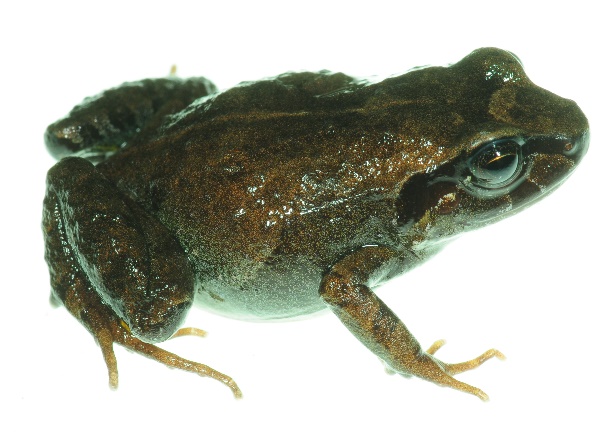 | 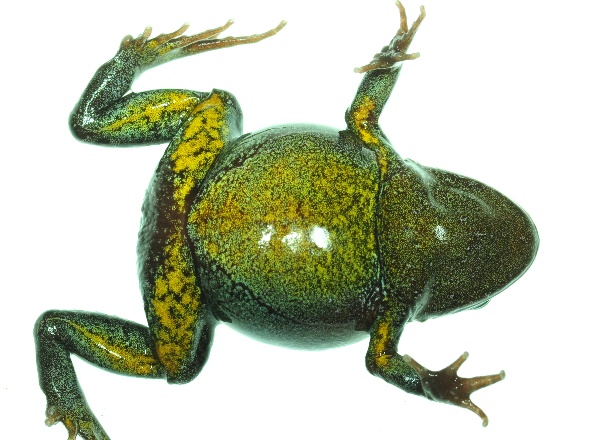 |

.

| PARATYPE (male) | CORBIDI 16497 (AC120.15) |
| --- | --- |
| 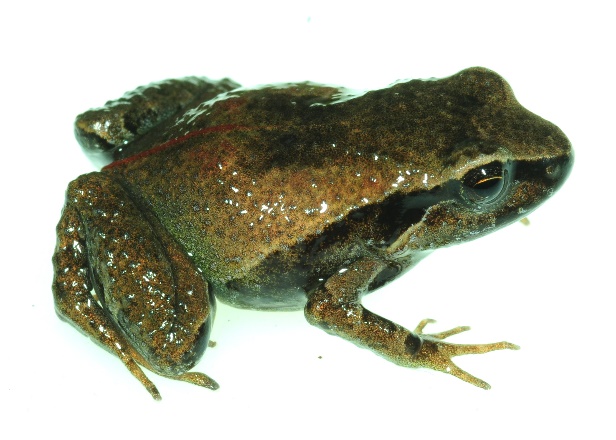 | 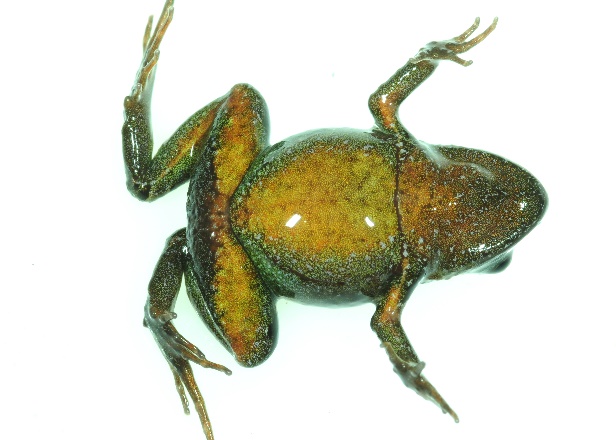 |
| PARATYPE (male) | CORBIDI 16496 (AC121.15) |
| 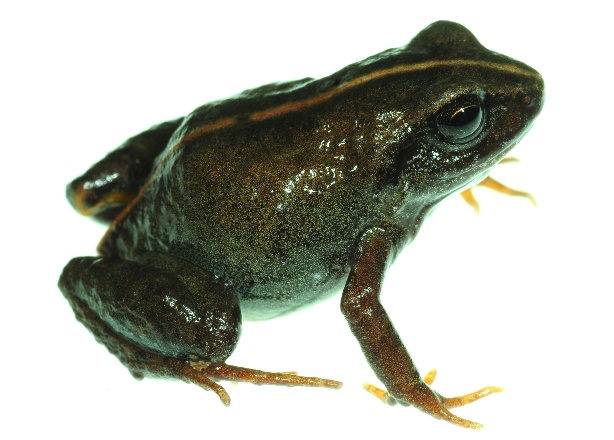 | 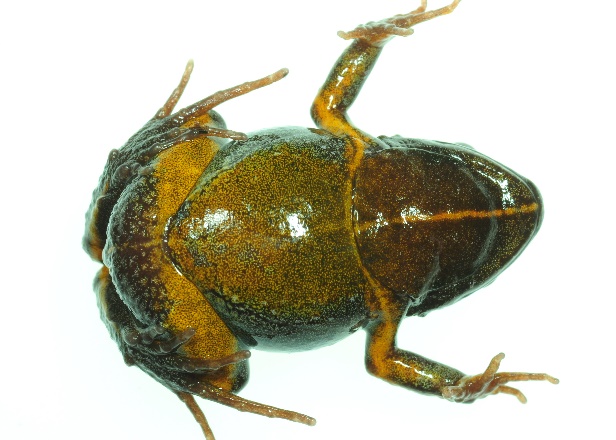 |
| PARATYPE (female) | MHNC 14661 (AC128.15) |
| 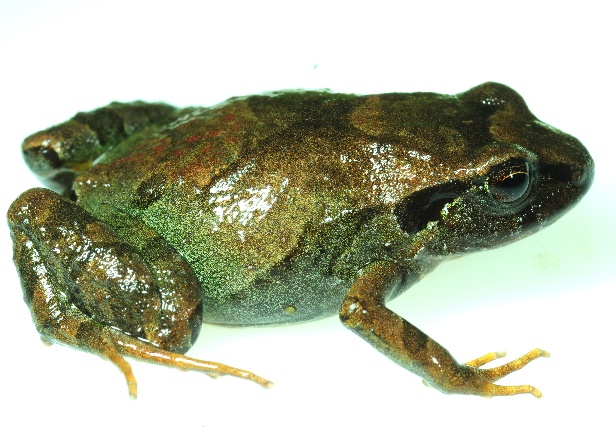 | 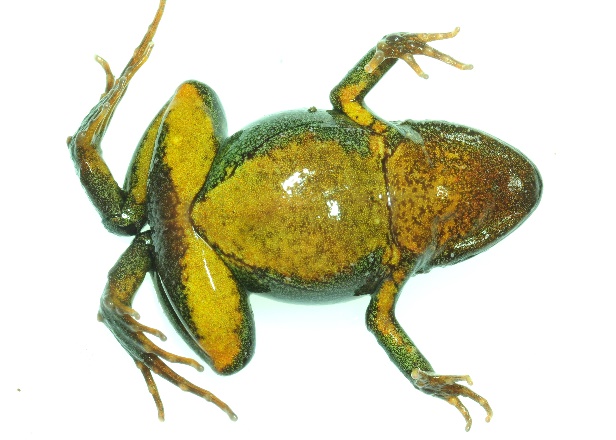 |
| PARATYPE (female) | CORBIDI 16500 (AC97.15) |
| 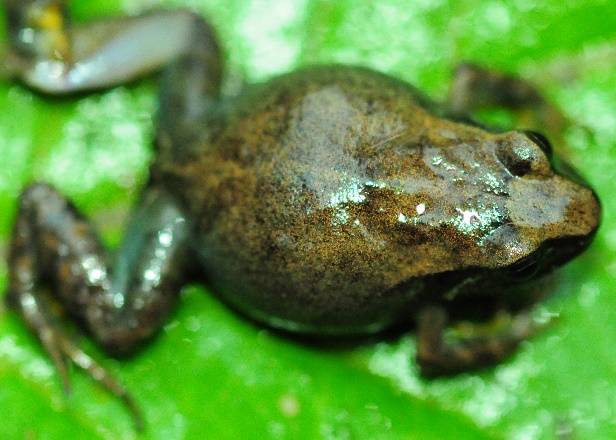 | 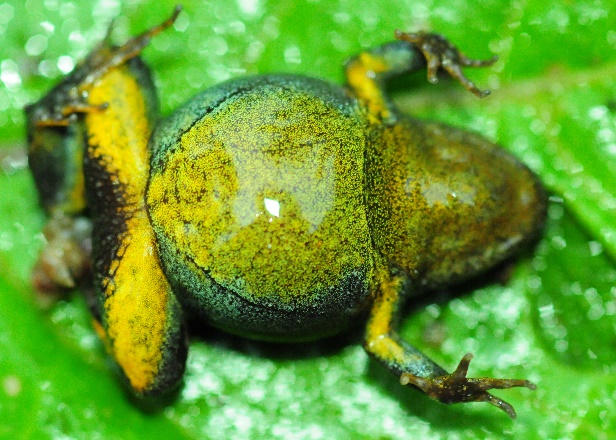 |

.

| UNCOLLECTED male | 635.15 |
| --- | --- |
| 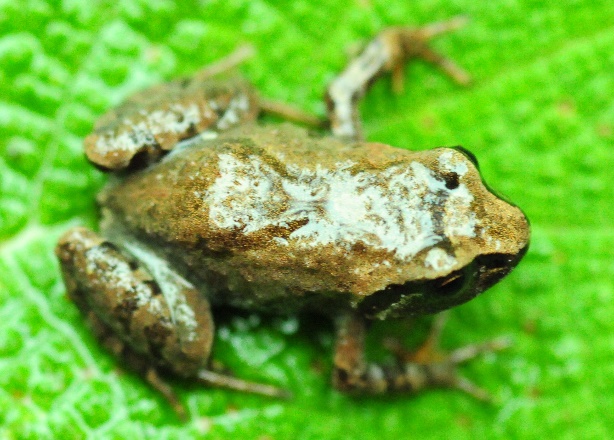 | 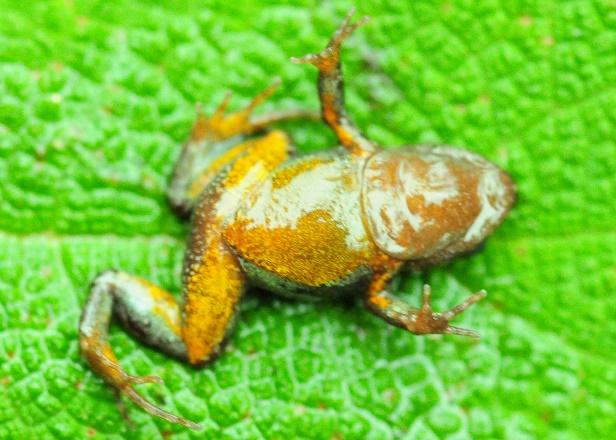 |
| UNCOLLECTED male | 636.15 |
| 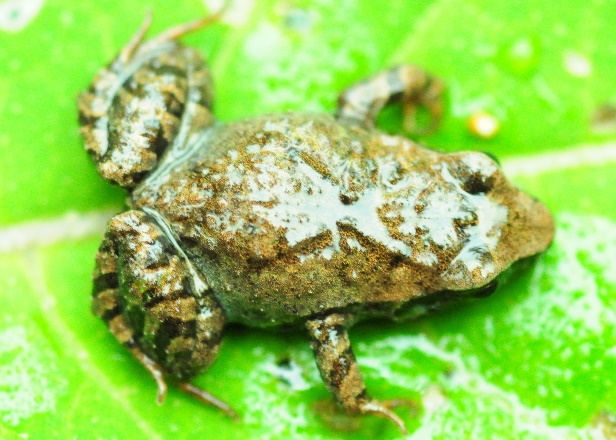 | 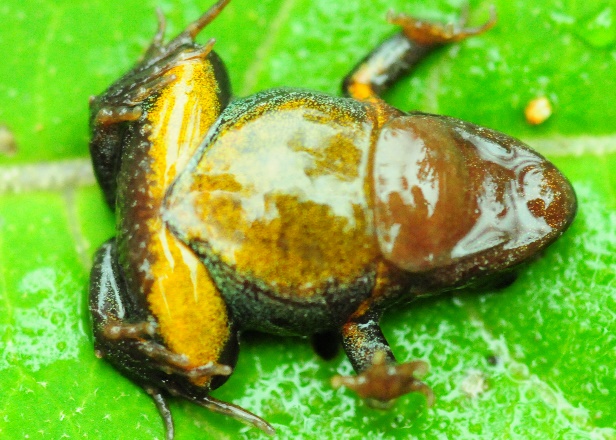 |
| UNCOLLECTED male | 639.15 |
| 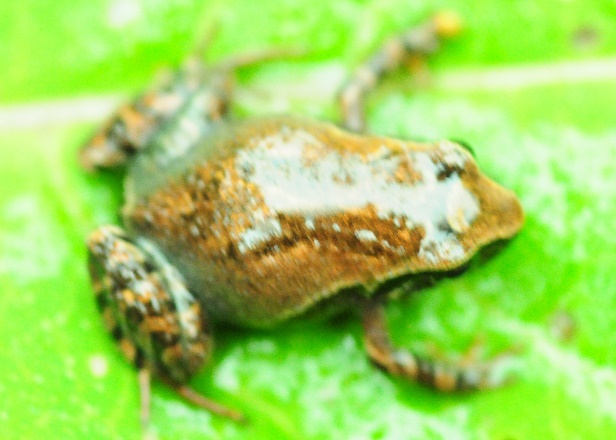 | 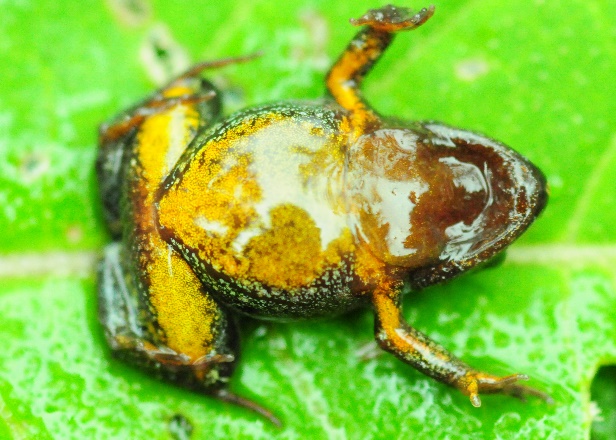 |
| UNCOLLECTED male | 640.15 |
| 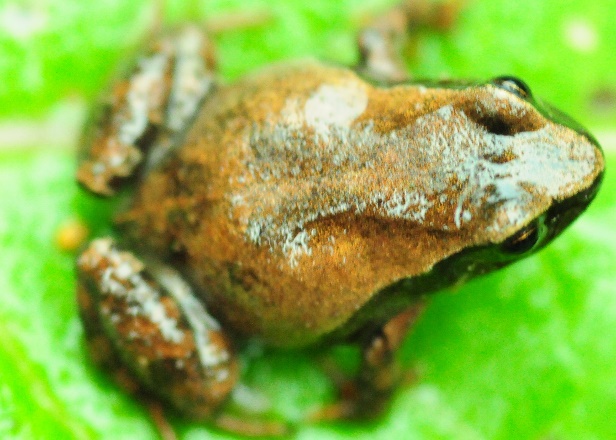 | 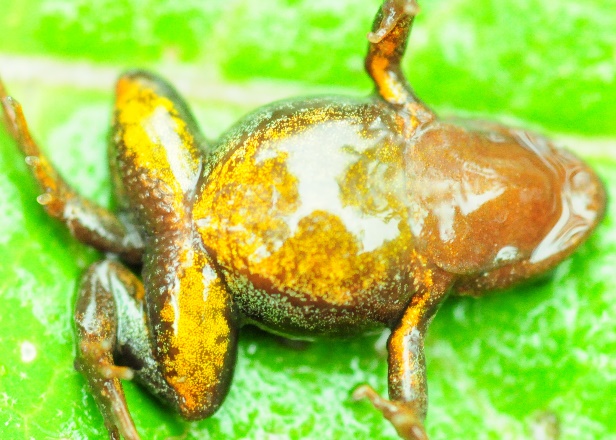 |

.

| UNCOLLECTED male | 1004.15 |
| --- | --- |
| 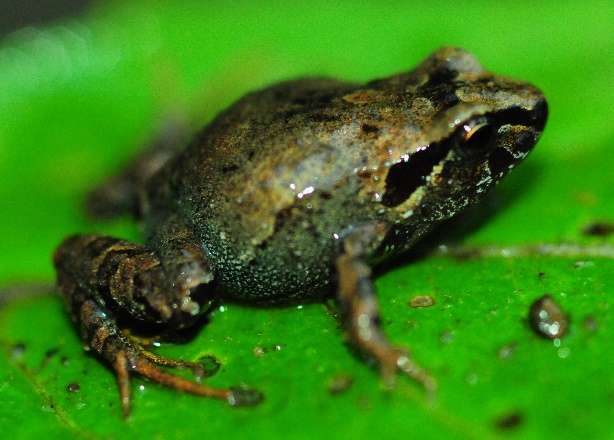 | 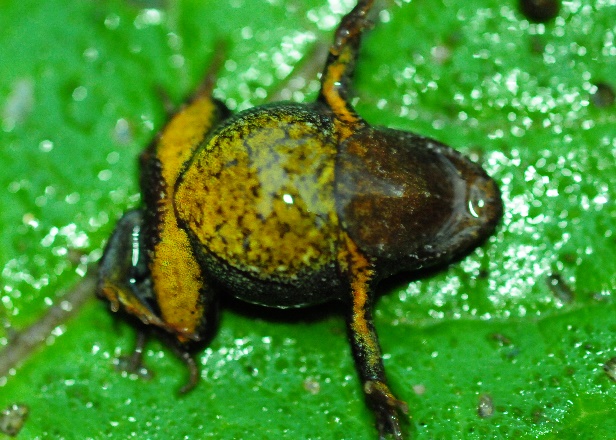 |
| UNCOLLECTED male | 1005.15 |
| 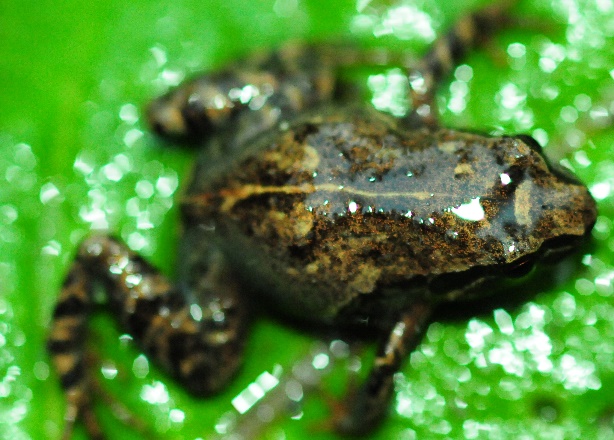 | 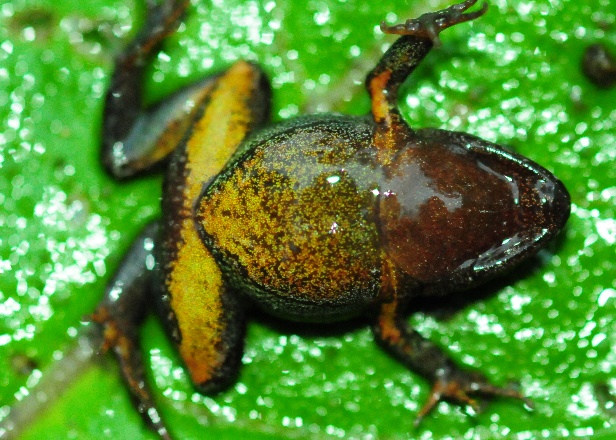 |
| UNCOLLECTED juvenile | 1006.15 |
| 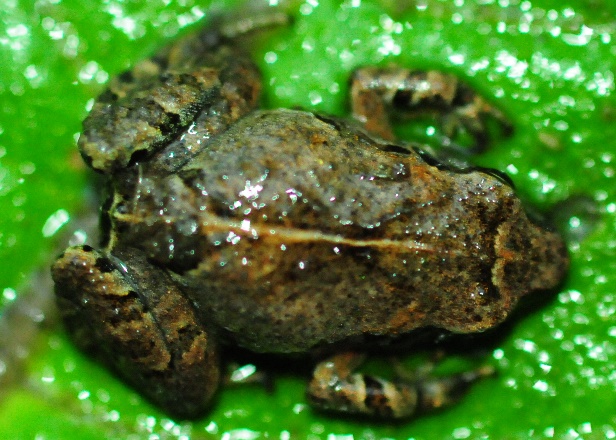 | 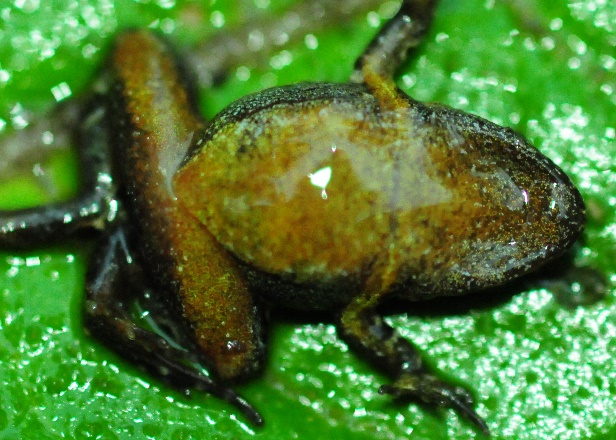 |
| UNCOLLECTED female | 1010.15 |
| 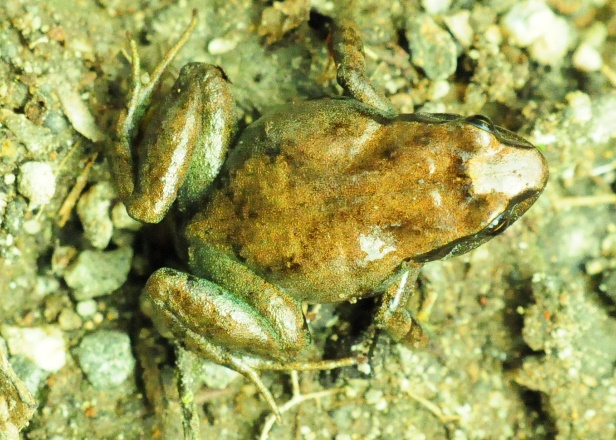 | 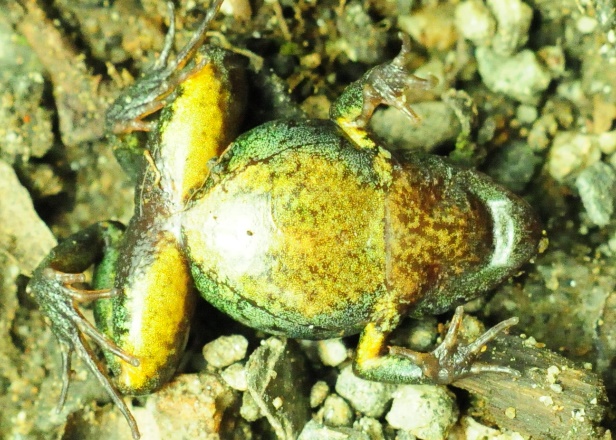 |

.

| UNCOLLECTED male | 1011.15 |
| --- | --- |
| 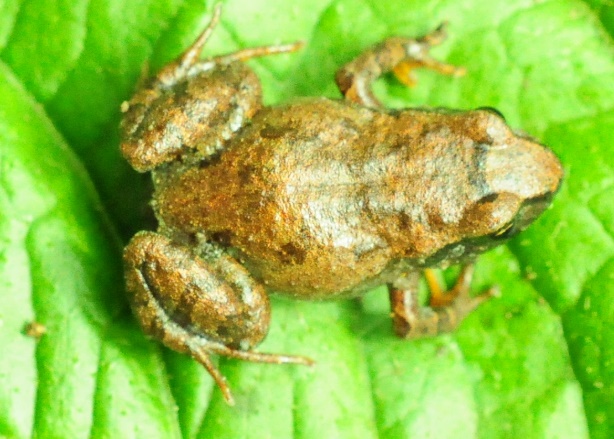 | 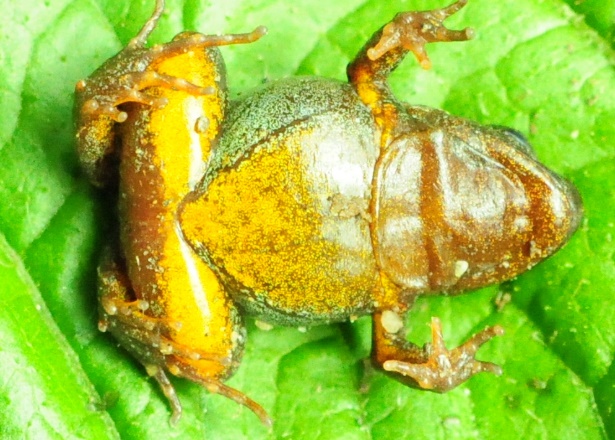 |
| UNCOLLECTED male | 1015.15 |
| 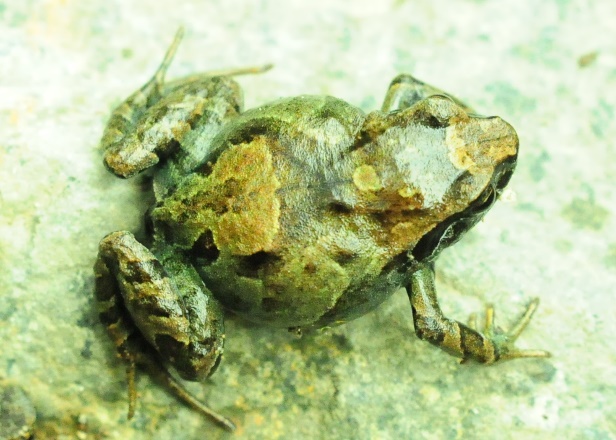 | 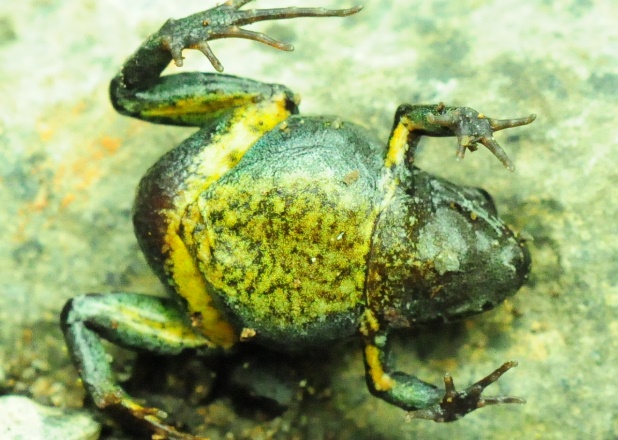 |
| UNCOLLECTED male | 1016.15 |
| 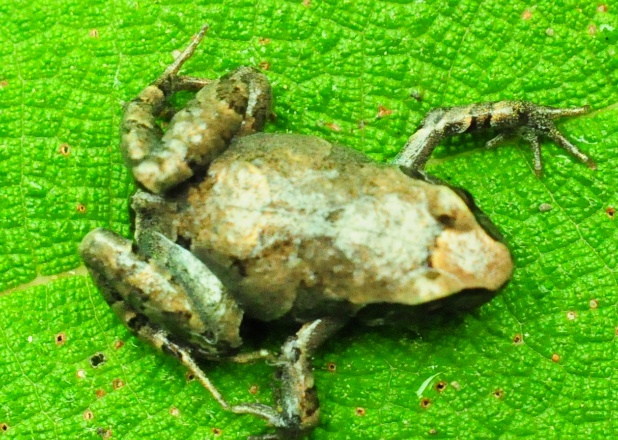 | 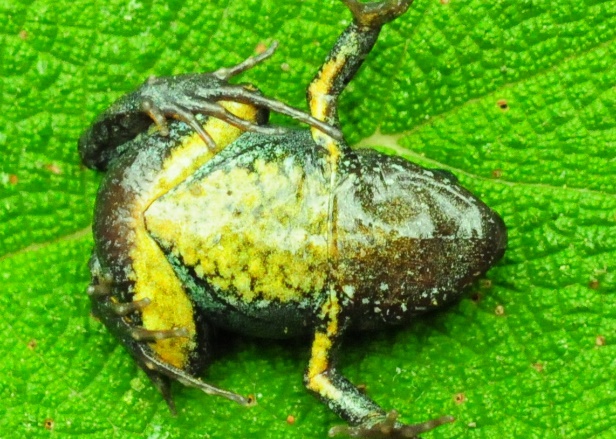 |
| UNCOLLECTED male | 1017.15 |
| 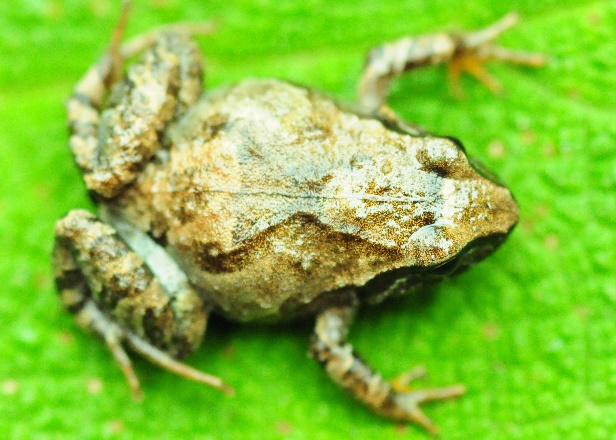 | 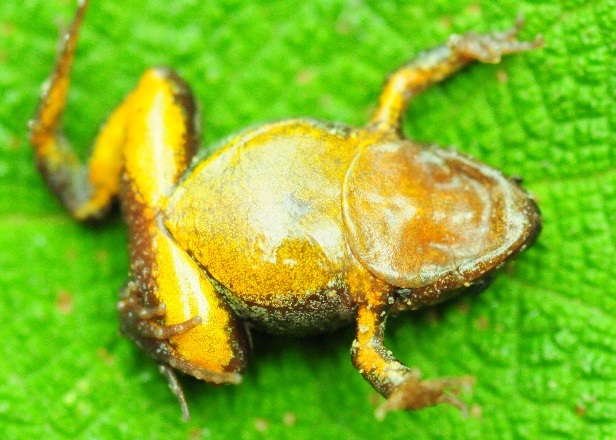 |

.

| UNCOLLECTED female | 1018.15 |
| --- | --- |
| 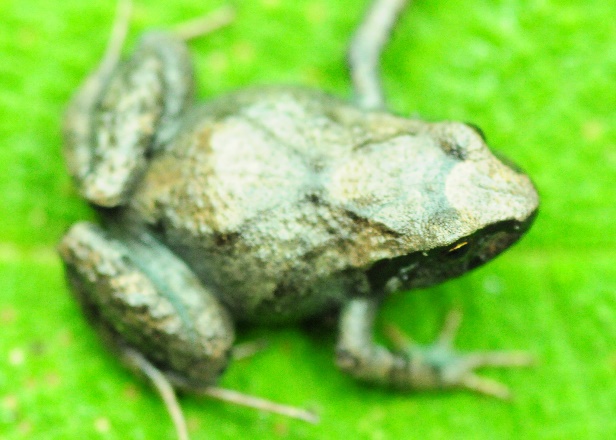 | 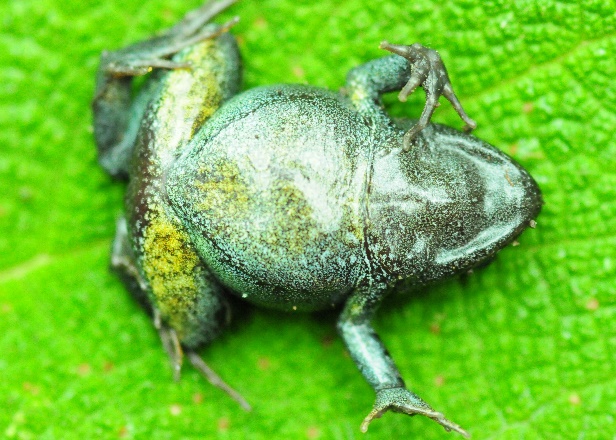 |
| UNCOLLECTED male | 1019.15 |
| 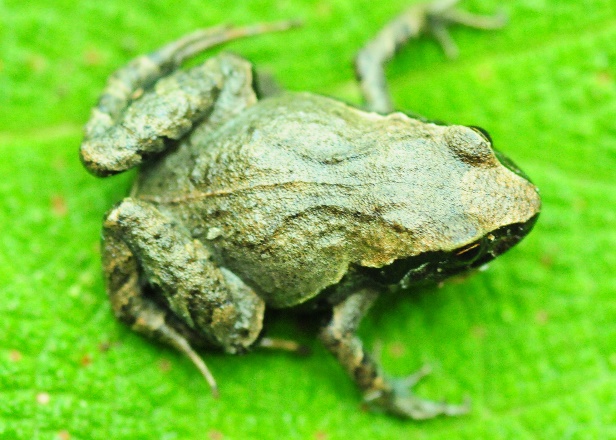 | 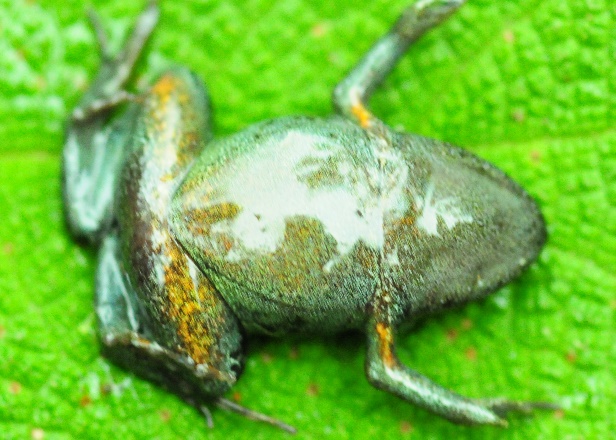 |
| UNCOLLECTED male | 1065.15 |
| 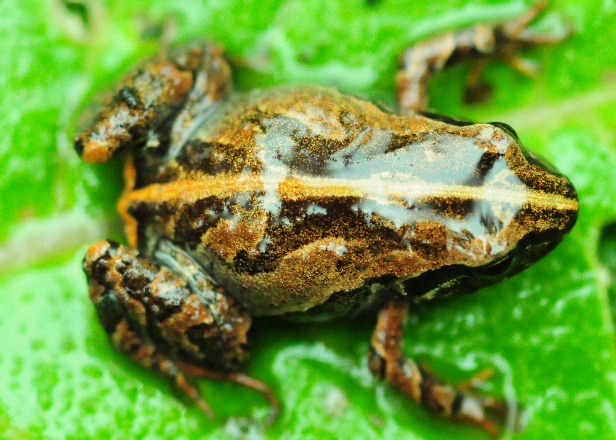 | 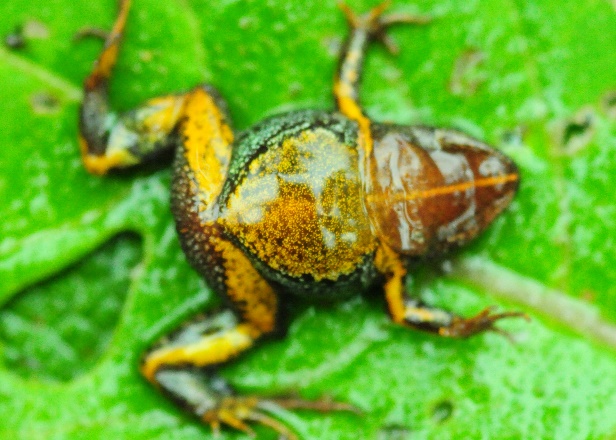 |
| UNCOLLECTED male | 1066.15 |
| 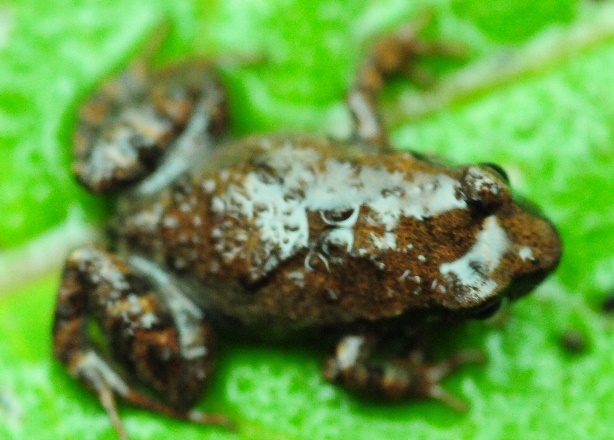 | 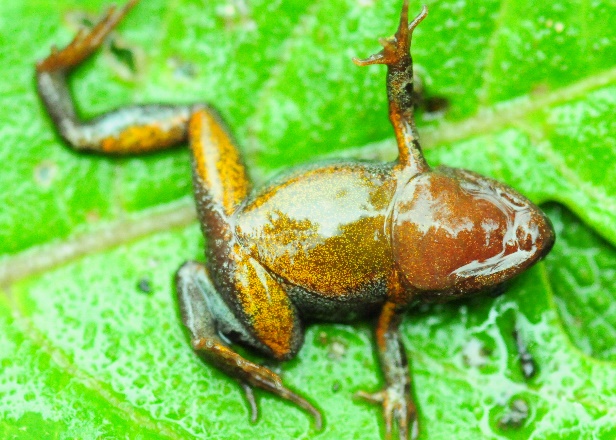 |

.

| UNCOLLECTED male | 1067.15 |
| --- | --- |
| 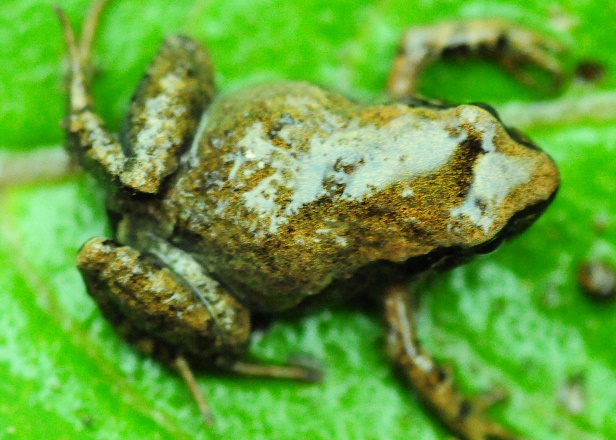 | 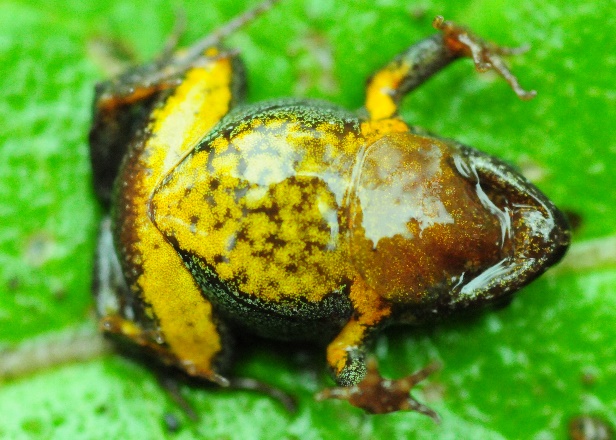 |
